# Lower Limit of Percolation Threshold on Square Lattice with Complex Neighborhoods

**DOI:** 10.3390/e27040361

**Published:** 2025-03-29

**Authors:** Antoni Piotr Ciepłucha, Marcin Utnicki, Maciej Wołoszyn, Krzysztof Malarz

**Affiliations:** Faculty of Physics and Applied Computer Science, AGH University, al. Mickiewicza 30, 30-059 Kraków, Polandwoloszyn@agh.edu.pl (M.W.)

**Keywords:** random site percolation, percolation threshold, complex neighborhoods, computer simulation

## Abstract

In this paper, the 60-year-old concept of long-range interaction in percolation problems introduced by Dalton, Domb and Sykes is reconsidered. With Monte Carlo simulation—based on the Newman–Ziff algorithm and the finite-size scaling hypothesis—we estimate 64 percolation thresholds for a random site percolation problem on a square lattice with neighborhoods that contain sites from the seventh coordination zone. The percolation thresholds obtained range from 0.27013 (for the neighborhood that contains only sites from the seventh coordination zone) to 0.11535 (for the neighborhood that contains all sites from the first to the seventh coordination zone). Similarly to neighborhoods with smaller ranges, the power-law dependence of the percolation threshold on the effective coordination number with an exponent close to −1/2 is observed. Finally, we empirically determine the limit of the percolation threshold on square lattices with complex neighborhoods. This limit scales with the inverse square of the mean radius of the neighborhood. The boundary of this limit is touched for threshold values associated with extended (compact) neighborhoods.

## 1. Introduction

Percolation [1,2,3,4] (see References [5,6] for recent reviews) is one of the core topics in statistical physics, providing the possibility to look at the critical phenomena occurring at phase transition solely on a geometrical basis, without sample heating, cooling, inserting in a magnetic field, etc. Some problems with respect to percolation can be treated analytically [7,8,9,10,11,12], but most studies are computational.

Originating from works by Broadbent and Hammersley [13,14] devoted to rheology (and still applied there [15,16,17,18]), it quickly found plenty of applications in physics, including in determining the magnetic [19,20,21,22,23] and electric [24,25,26] properties of solids or in nano-engineering [27].

However, the application of percolation theory is not restricted to physics alone. Examples (mainly two-dimensional systems [28,29,30,31,32]) can also be found in the fields of epidemiology [33,34,35], forest fires [36,37,38,39,40], agriculture [41,42,43], urbanization [44,45], materials chemistry [46], sociology [47], psychology [48], information transfer [49], finances [50] and dentistry [51].

Further, some effort was put into studying percolation on cubic (d=3 [52,53,54,55,56,57]) and hyper-cubic lattices, and also for non-physical dimensions (d=4 [52,58,59,60], d=5 [52,60,61] and d≥6 [52,62,63]). Simultaneously, the complex [6,49,64], distorted [65,66,67] and fractal [68] networks were studied.

Recently, the 60-year-old concept of long-range interaction [69,70] has sparked renewed interest. The neighborhoods that contain sites that are not nearest neighbors on an assumed lattice are called extended neighborhoods (when they are compact) or complex (when they are non-compact, that is, when they contain ‘wholes’)—to keep the nomenclature from Reference [71]. Many papers were devoted to studies of the percolation thresholds on the extended [49,59,61,72,73,74] and the complex [57,58,71,75,76,77,78,79,80,81,82] neighborhoods.

The particular neighborhood names—now keeping the convention proposed in Reference [72]—are combinations of alphanumeric strings. The first two letters identify the underlying lattice (for example, sq for square, tr for triangular, hc for honeycomb and sc for simple cubic lattices), then they are accompanied by a numerical string indicating the coordination zones which constitute the neighborhood. In this convention, the von Neumann neighborhood on the square lattice (with only the nearest neighbors) is called sq-1, while Moore’s neighborhood on the square lattice (containing sites from the first and the second coordination zones) is called sq-1,2.

In this paper, we are closer to the theoretical studies of percolation phenomena than to their application. That is, our studies focus on the influence of long-range interactions on the percolation threshold $p_{c}$. The percolation threshold is the equivalent of the critical point in phase transition phenomena. For a random site percolation problem, we deal with the nodes of the lattice that are occupied (with probability *p*) or empty (with probability 1−p). Occupied sites, in an assumed neighborhood, are considered to form a cluster. Depending on the sites’ occupation probability *p*, such a cluster may span (or not) the system edges. The percolation threshold $p_{c}$ is such an occupation probability *p* where, for $p<p_{c}$, a spanning cluster is absent (and the system behaves as an insulator), while, for $p>p_{c}$, a spanning cluster is present (and the system behaves as a conductor). Thus, $p_{c}$ separates two phases, isolating and conducting, and, at $p=p_{c}$, the (second-order) phase transition takes place.

**Figure 1:**
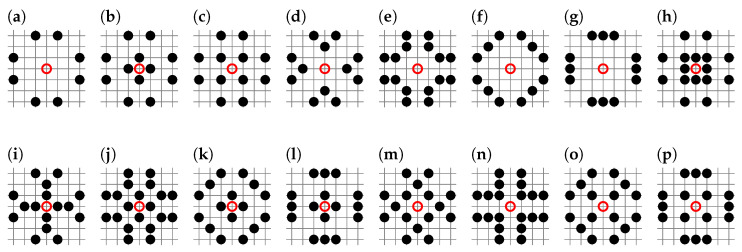

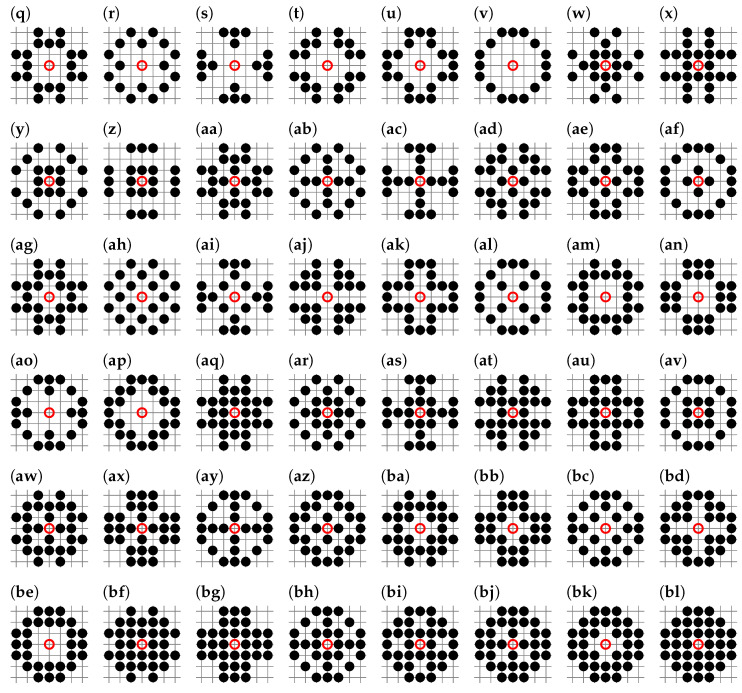
Neighborhoods of the site shown as the red circle on square lattice containing sites from the 7th coordination zone: (**a**) sq-7, (**b**) sq-1,7, (**c**) sq-2,7, (**d**) sq-3,7, (**e**) sq-4,7, (**f**) sq-5,7, (**g**) sq-6,7, (**h**) sq-1,2,7, (**i**) sq-1,3,7, (**j**) sq-1,4,7, (**k**) sq-1,5,7, (**l**) sq-1,6,7, (**m**) sq-2,3,7, (**n**) sq-2,4,7, (**o**) sq-2,5,7, (**p**) sq-2,6,7, (**q**) sq-3,4,7, (**r**) sq-3,5,7, (**s**) sq-3,6,7, (**t**) sq-4,5,7, (**u**) sq-4,6,7, (**v**) sq-5,6,7, (**w**) sq-1,2,3,7, (**x**) sq-1,2,4,7, (**y**) sq-1,2,5,7, (**z**) sq-1,2,6,7, (**aa**) sq-1,3,4,7, (**ab**) sq-1,3,5,7, (**ac**) sq-1,3,6,7, (**ad**) sq-1,4,5,7, (**ae**) sq-1,4,6,7, (**af**) sq-1,5,6,7, (**ag**) sq-2,3,4,7, (**ah**) sq-2,3,5,7, (**ai**) sq-2,3,6,7, (**aj**) sq-2,4,5,7, (**ak**) sq-2,4,6,7, (**al**) sq-2,5,6,7, (**am**) sq-3,4,5,7, (**an**) sq-3,4,6,7, (**ao**) sq-3,5,6,7, (**ap**) sq-4,5,6,7, (**aq**) sq-1,2,3,4,7, (**ar**) sq-1,2,3,5,7, (**as**) sq-1,2,3,6,7, (**at**) sq-1,2,4,5,7, (**au**) sq-1,2,4,6,7, (**av**) sq-1,2,5,6,7, (**aw**) sq-1,3,4,5,7, (**ax**) sq-1,3,4,6,7, (**ay**) sq-1,3,5,6,7, (**az**) sq-1,4,5,6,7, (**ba**) sq-2,3,4,5,7, (**bb**) sq-2,3,4,6,7, (**bc**) sq-2,3,5,6,7, (**bd**) sq-2,4,5,6,7, (**be**) sq-3,4,5,6,7, (**bf**) sq-1,2,3,4,5,7, (**bg**) sq-1,2,3,4,6,7, (**bh**) sq-1,2,3,5,6,7, (**bi**) sq-1,2,4,5,6,7, (**bj**) sq-1,3,4,5,6,7, (**bk**) sq-2,3,4,5,6,7, (**bl**) sq-1,2,3,4,5,6,7.

Here, we calculate the percolation thresholds $p_{c}$ for random site percolation in the square lattice for neighborhoods containing sites from the seventh coordination zone. There are 64 such neighborhoods, from sq-7 to sq-1,2,3,4,5,6,7, and they are presented in Figure 1.

The paper is organized as follows: In Section 2, we recall the finite-size scaling hypothesis, present basics of the effective Newman–Ziff Monte Carlo algorithm for the percolation problem, define the effective coordination number and give some details regarding the technicalities of the computations. In Section 3, we show the results of the Monte Carlo simulations, which allow for the estimation of 64 values of $p_{c}$ for various neighborhoods together with their geometrical characteristics, such as their total and effective coordination numbers. Finally, Section 4 is devoted to a discussion of the obtained results.

## 2. Methods

### 2.1. Finite-Size Scaling Hypothesis

In the vicinity of the critical point $x_{c}$, many observables *X* obey the finite-size scaling [1,83,84] relation

(1) $$X\left(x;L\right)=L^{-\varepsilon_{1}}\cdot F\left(\left(x-x_{c}\right)L^{\varepsilon_{2}}\right),$$

where *x* reflects the level of system disorder, F is the scaling function (usually analytically unknown) and $\varepsilon_{1,2}$ are universal exponents. These exponents depend only on the physical dimension of the system *d*. Knowing $\varepsilon_{1,2}$ and plotting X(x;L) leads to the collapse of many curves (for various *L*) into a single one.

At the critical point $x=x_{c}$, the value of

(2) $$X\left(x=x_{c};L\right)\cdot L^{\varepsilon_{1}}=F\left(0\right)$$

does not depend on the size of the system *L*, which opens a computationally feasible way of searching for both the critical point $x_{c}$ and the critical exponents $\varepsilon_{1,2}$.

For the random site percolation problem, the probability

(3) $$P_{\max}=S_{\max}/L^{2},$$

that the randomly chosen site belongs to the largest cluster (with size $S_{\max}$) may play the role of the quantity *X*. Then, the exponents $\varepsilon_{1,2}$ are known analytically, and $\varepsilon_{1}=5/48$ while $\varepsilon_{2}=3/4$.

### 2.2. Efficient Newman–Ziff Algorithm

To calculate the probability $P_{\max}$ mentioned above, we use the Newman–Ziff algorithm [85]. The algorithm is fast as it is based on the concept of calculating desired quantities only after adding a single occupied site to the system that so far exists. This allows us to construct the dependence of X(n), where *n* is the number of occupied sites.

The second part of the algorithm is the transformation of X(n) values from the space of the integer number *n* of occupied sites to X(p) in the space of the real numbers of probabilities *p* of site occupation

(4) $$X\left(p;N\right)=\sum_{n=0}^{N}\bar{X}\left(n;N\right)B\left(n;N,p\right),$$

where $N=L^{2}$ stands for the size of the system. This conversion requires knowing the Bernoulli (binomial) probability distribution

(5) B(n;N,p)=Nnpn(1−p)N−n

and Reference [85] provides an efficient way of performing recursive calculation of the binomial distribution coefficients in Equation (5).

We implement the Newman–Ziff algorithm as a computer program written in C language. This requires modification of the original boundaries() procedure provided in Reference [85] beyond the nearest neighbors. In Listing A1, available in Appendix A, we show an example of such a modification for the sq-7 neighborhood presented in Figure 1a.

### 2.3. Effective Coordination Number

The values of $p_{c}$ usually degenerate strongly with respect to the neighborhood coordination number *z*. Degeneration means that, for a given coordination number (number of sites in the neighborhood), many various values of $p_{c}$ are associated with this coordination number *z*. This degeneration may be removed (at least partially) when, instead of dealing with the coordination number *z*, we use the effective coordination number

(6) $$\zeta =\sum_{i}z_{i}r_{i},$$

where $z_{i}$ and $r_{i}$ are the number of sites and their distance from the central site in the neighborhood in the *i*-th coordination zone [81,82], respectively. Very recently, it was shown that, for square, honeycomb and triangular lattices,

(7) $$p_{c}\left(\zeta \right)\propto \zeta^{-w}$$

and exponent w≈1/2 [71].

## 3. Results

**Figure 2:**
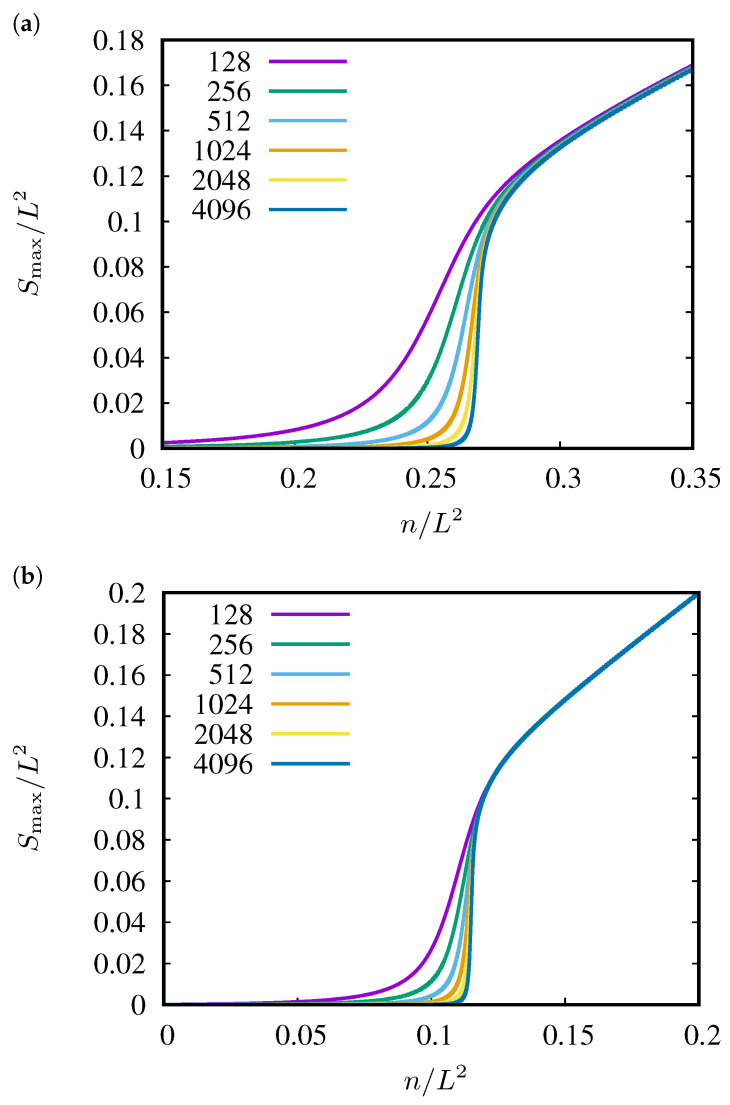
Examples of dependencies of $S_{\max}/L^{2}$ versus $n/L^{2}$ for various linear system sizes: L=128, 256, 512, 1024, 2048 and 4096 from top to bottom. (**a**) sq-7, (**b**) sq-1,2,3,4,5,6,7.

In Figure 2, we present the dependencies of the probabilities $P_{\max}\left(p\right)$ of belonging to the largest cluster on sites occupation probability $p=n/L^{2}$. $P_{\max}$ values are defined by geometrical probability as the size of the largest cluster $S_{\max}$ per the system size $L^{2}$. The results are averaged over $R=10^{5}$ realizations of systems that contain $128^{2}$, $256^{2}$, $512^{2}$, $1024^{2}$, $2048^{2}$ and $4096^{2}$ sites. The examples correspond to the neighborhoods sq-7 (Figure 2a) and sq-1,2,3,4,5,6,7 (Figure 2b).

**Figure 3:**
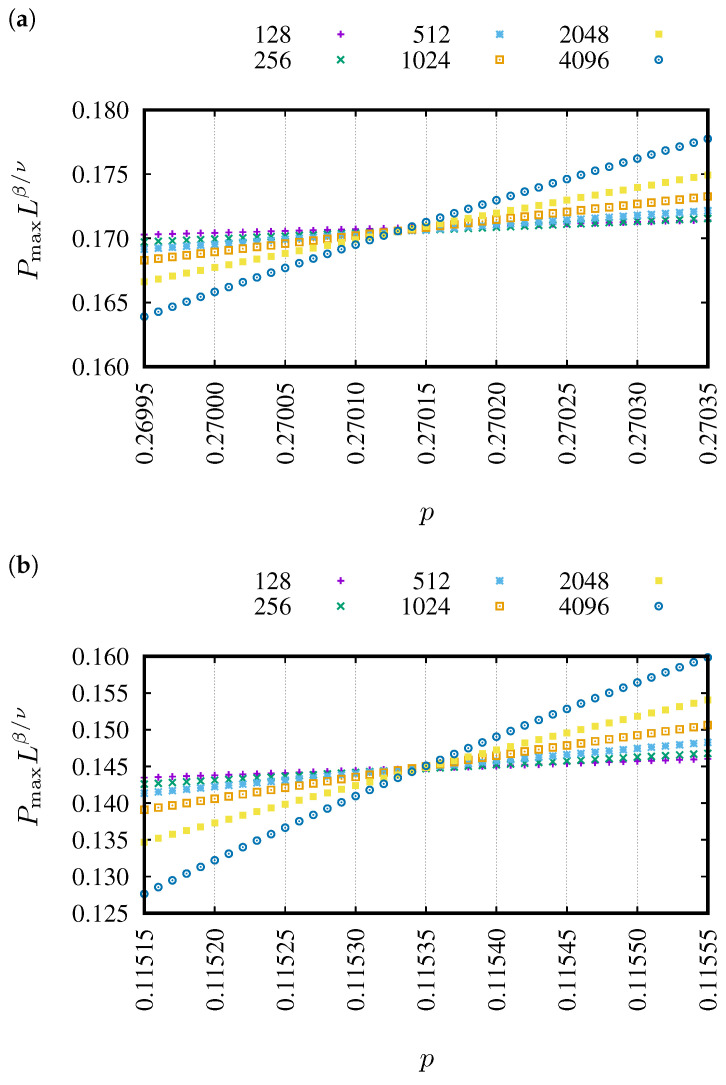
Examples of dependencies of $P_{\max}L^{\beta /\nu }$ versus *p* for various linear system sizes: L=128, 256, 512, 1024, 2048 and 4096 from top to bottom. (**a**) sq-7, $p_{c}$ (sq-7) = 0.27013, (**b**) sq-1,2,3,4,5,6,7, $p_{c}$ (sq-1,2,3,4,5,6,7) = 0.11535.

In Figure 3, examples of dependencies of $P_{\max}L^{\beta /\nu }$ versus *p* are presented for various linear system sizes *L*. The examples correspond to the neighborhoods sq-1 (Figure 3a) and sq-1,2,3,4,5,6,7 (Figure 3b). These dependencies for 64 neighborhoods containing sites from the seventh coordination zone are presented in Figure 1 in Reference [86]. The common cross-point for various lattice sizes predicts the percolation threshold $p_{c}$.

The evaluated percolation thresholds $p_{c}$, the total (*z*) and the effective (ζ) coordination numbers for various neighborhoods are collected in Table 1.

**Figure 4:**
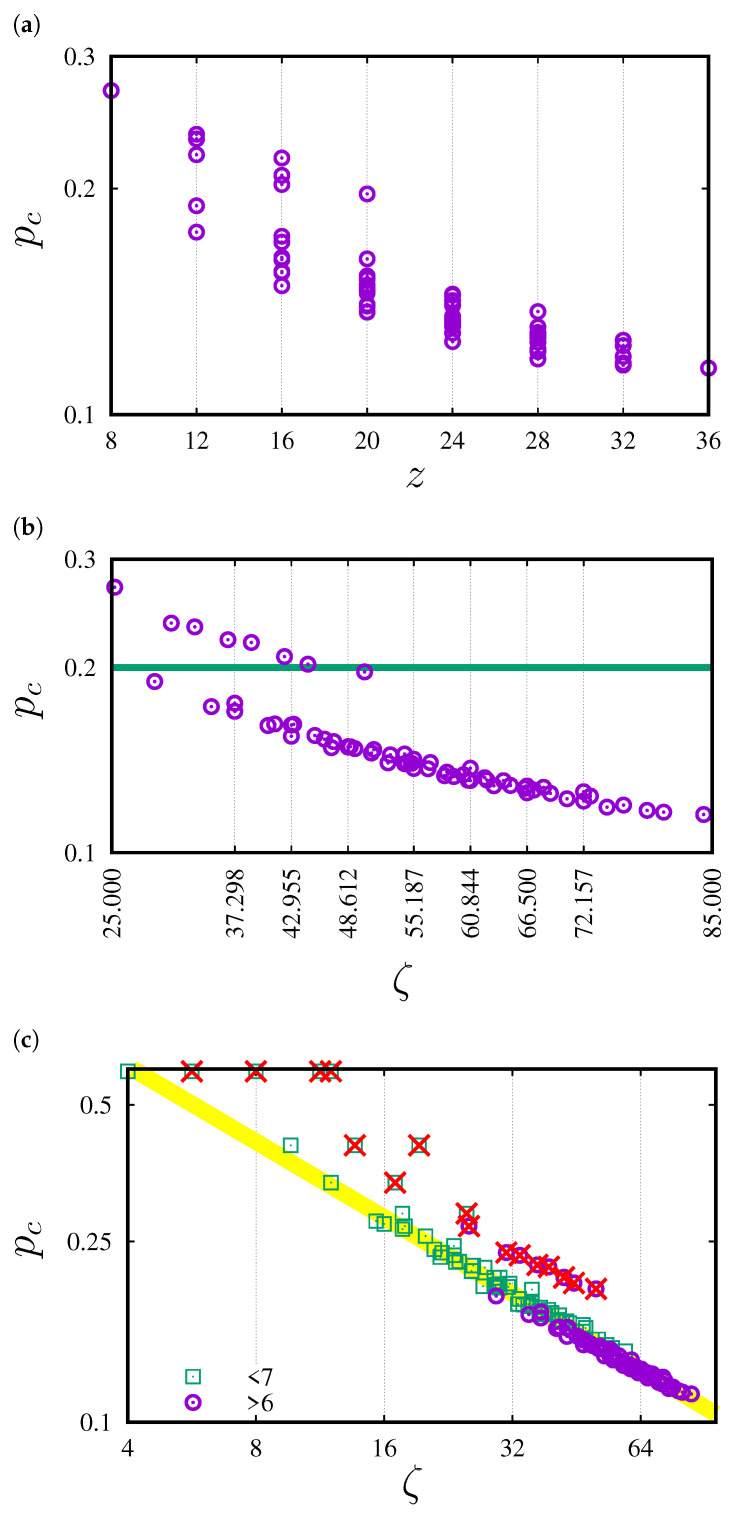
Dependencies of $p_{c}$ on (**a**) the total (*z*) and (**b**) effective (ζ) coordination number for neighborhoods presented in Figure 1. (**c**) $p_{c}\left(\zeta \right)$ with additional data also for neighborhoods (marked by □) containing sites up to the 6th coordination zone (from Reference [71] and references therein). The inflated neighborhoods (marked by ×) are excluded from the fitting procedure. The least-squares fit gives exponent w≈0.5552(42).

In Figure 4, the dependencies of the percolation threshold $p_{c}$ on the total (*z*, Figure 4a) and effective (ζ, Figure 4b) values of coordination numbers are presented. Figure 4c shows $p_{c}\left(\zeta \right)$ with additional data also for neighborhoods containing sites up to the sixth coordination zone [71]. The inflated neighborhoods (marked by ×) are excluded from the fitting procedure. The data are accompanied by the least-squares method fit to the power law (7). The estimated value of the exponent w≈0.5552(42).

## 4. Discussion

In this paper, the estimation of $p_{c}$ for random site percolation on a square lattice for neighborhoods containing sites from the seventh coordination zone is presented. Monte Carlo simulation with $R=10^{5}$ system realizations for the system sizes from $128^{2}$ to $4096^{2}$ sites allowed us to estimate the percolation thresholds to $10^{-5}$ accuracy.

The obtained percolation thresholds range from 0.27013 (for the neighborhood that contains solely sites from the seventh coordination zone) to 0.11535 (for the neighborhood that contains all sites from the first to the seventh coordination zone). The latter agrees perfectly (with the accuracy obtained here, that is $10^{-5}$) with the earlier results of the extensive Monte Carlo simulation (with L=16,384 and $R>3\times 10^{8}$ independent samples produced for each lattice) where *p_c_*(sq-1,2,3,4,5,6,7) ≈0.1153481(9) [72].

**Figure 5:**
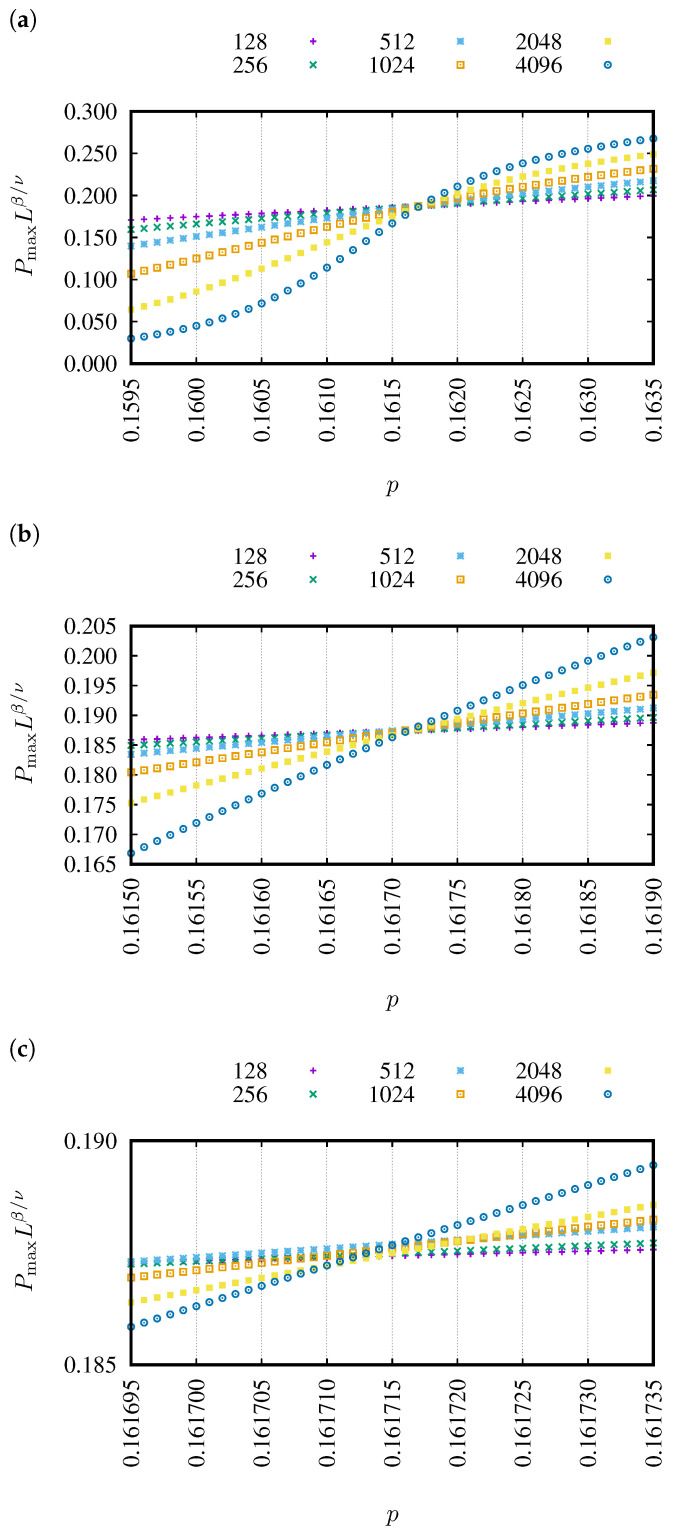
Dependencies $L^{\beta /\nu }\cdot P_{\max}\left(p\right)$ for various values of the separation Δp: (**a**) $10^{-4}$, (**b**) $10^{-5}$, (**c**) $10^{-6}$. With an assumed number of repetitions of simulations ($R=10^{5}$), value $\Delta p=10^{-6}$ is insufficient to see a clear “single crossing point” and to identify $p=p^{*}$.

Capturing the intersection of the rescaled probabilities of belonging to the largest cluster $L^{\beta /\nu }\cdot P_{\max}\left(p\right)$ numerically for finite systems is quite challenging as the curves for various *L* values rather seldom intersect exactly at one point (as predicted theoretically by Equation (2)). Also, in our case, as shown in Figure 3, we do not have the intersection of the $L^{\beta /\nu }\cdot P_{\max}\left(p\right)$ curves for different *L* values occurring at one point, but rather we can easily identify the value of $p^{*}$ for which the mutual squared differences

$$\delta^{2}\left(p\right)=\sum_{i,j}{\left[L_{i}^{\beta /\nu }\cdot P_{\max}\left(p;L_{i}\right)-L_{j}^{\beta /\nu }\cdot P_{\max}\left(p;L_{j}\right)\right]}^{2}\;$$

between the $L^{\beta /\nu }\cdot P_{\max}\left(p\right)$ values—for all studied *L* values—are the smallest. This value of $p^{*}$ for which the mutual squared differences $\delta^{2}\left(p\right)$ reach their minimum estimates the percolation threshold $p_{c}\approx p^{*}$ [87]. The convolution (4) can be performed for any arbitrary value of *p*, but the secret to achieving such a small $\delta^{2}$ value that one has the impression it tends to zero lies in the reasonable assumption of the separation Δp with which we scan *p* values. In Figure 5, we show examples of $L^{\beta /\nu }\cdot P_{\max}\left(p\right)$ for $\Delta p=10^{-4}$, $10^{-5}$ and $10^{-6}$. As can be seen, these dependencies allow us to easily indicate the “intersection point” for $\Delta p=10^{-4}$ (Figure 5a) and $10^{-5}$ (Figure 5b). That is, just by inspection, even without calculating $\delta^{2}$, one can see a higher dispersion of the values $L^{\beta /\nu }\cdot P_{\max}\left(p\right)$ for the considered values of *L* and thus a larger $\delta^{2}\left(p\right)$ at points $p=(p^{*}-\Delta p)$ and $p=(p^{*}+\Delta p)$ than at $p=p^{*}$. This means that the true value of $p_{c}$ is somewhere inside the interval $\left(p^{*}-\Delta p,p^{*}+\Delta p\right)$.

On the other hand, for $\Delta p=10^{-6}$ (see Figure 5c), we cannot easily identify the “intersection point”. The reason for this lies in too weak statistics, i.e., number *R* of the simulation repetition is too small. The precision of determining the value of $P_{\max}\left(n\right)$—i.e., $\bar{X}\left(n;N\right)$ in Equation (4)—is 1/R and affects the selection of the Δp value, which allows observation of a clear $\delta^{2}\left(p\right)$ minimum for $p=p^{*}$, with a simultaneous clear spread of points $L^{\beta /\nu }\cdot P_{\max}\left(p\right)$ for various *L* values at $p=p^{*}-\Delta p$ and $p=p^{*}+\Delta p$. Hence, we consider $\Delta p=10^{-5}$ as the uncertainty of the determined $p_{c}$. Moreover, plotting $L^{\beta /\nu }\cdot P_{\max}\left(p\right)$ for a finite *L* according to Equation (1) allows us to eliminate, at least partially, the effect of finite sizes on the accuracy of determining $p_{c}$.

**Figure 6:**
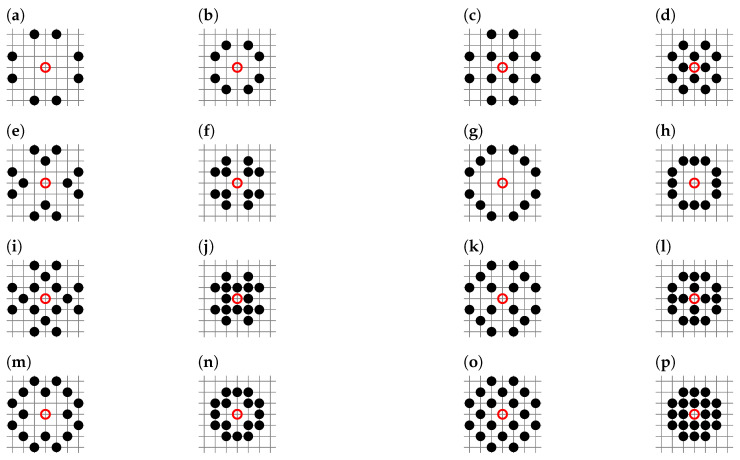
Inflated neighborhoods (**a**) sq-7, (**c**) sq-2,7, (**e**) sq-3,7, (**g**) sq-5,7, (**i**) sq-2,3,7, (**k**) sq-2,5,7, (**m**) sq-3,5,7, (**o**) sq-2,3,5,7 and their lower indexed partners (**b**) sq-4, (**d**) sq-1,4, (**f**) sq-2,4, (**h**) sq-3,4, (**j**) sq-1,2,4, (**l**) sq-1,3,4, (**n**) sq-2,3,4, (**p**) sq-1,2,3,4.

In Figure 4b, we can clearly observe two series of data, roughly for $p_{c}$ below and above 0.2. The latter corresponds to sq-7, sq-2,7, sq-3,7, sq-5,7, sq-2,3,7, sq-2,5,7, sq-3,5,7 and sq-2,3,5,7. These neighborhoods are the so-called inflated neighborhoods, which means that they have partners with lower indexed neighborhoods with the same $p_{c}$ and *z*. These partners are presented in Figure 6.

Similarly to neighborhoods with smaller ranges, the power-law dependence of the percolation threshold on the effective coordination number with exponent *w* close to −1/2 is observed. The power law is obtained after excluding $p_{c}$ of the inflated neighborhoods (marked by × in Figure 4c) from the fitting procedure.

Introducing the effective coordination number ζ partially eliminates the degeneration (see Figure 4b) strongly observed in the dependence on $p_{c}\left(z\right)$ (see Figure 4a). For $p_{c}\left(\zeta \right)$, we still observe this degeneration in several cases, such as

- ζ(SQ-1,3,7)=ζ(SQ-6,7);
- ζ(SQ-1,2,3,7)=ζ(SQ-2,6,7);
- ζ(SQ-1,3,5,7)=ζ(SQ-5,6,7);
- ζ(SQ-1,3,4,7)=ζ(SQ-4,6,7);
- ζ(SQ-1,2,3,4,7)=ζ(SQ-2,4,6,7);
- ζ(SQ-1,3,4,5,7)=ζ(SQ-4,5,6,7);
- ζ(SQ-1,2,3,4,5,7)=ζ(SQ-2,4,5,6,7).

This degeneration in distinguishing neighborhoods based only on the scalar variable can be resolved after normalization of ζ to the total number of sites in the neighborhood *z*. The fraction ζ/z is nothing else but the mean distance

(8) $$\bar{r}=\frac{1}{z}\sum_{i}z_{i}r_{i}$$

of sites in the neighborhood to the central site. We can call it the mean radius of the neighborhood.

**Figure 7:**
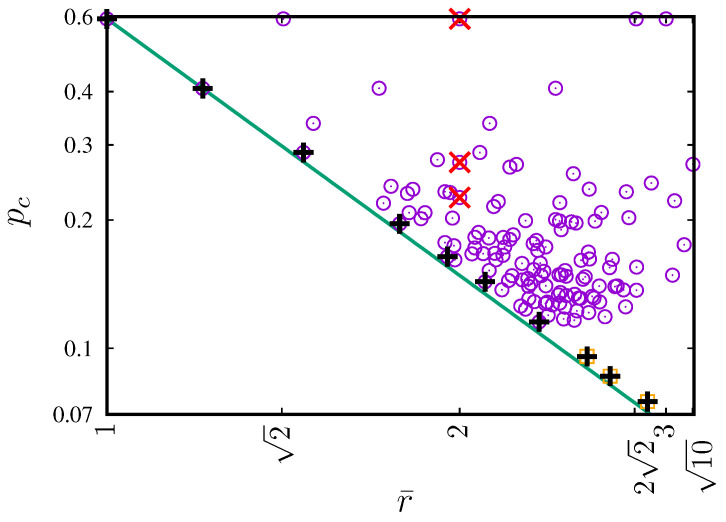
Dependence of percolation threshold $p_{c}$ on mean radius $\bar{r}$ of the neighborhoods. The mean radius $\bar{r}$ uniquely identifies neighborhoods except the three neighborhoods sq-3, sq-1,6 and sq-1,3,6, all with a mean radius equal to two. These three neighborhoods are marked by crosses (×). The open squares (□) correspond to the sq-1,2,3,4,5,6,7,8, sq-1,2,3,4,5,6,7,8,9, sq-1,2,3,4,5,6,7,8,9,10 neighborhoods (from Reference [73]). Pluses (+) show the $p_{c}$ associated with compact neighborhoods.

In Figure 7, we show the dependence $p_{c}$ on the mean radius $\bar{r}$ for the 131 neighborhoods that contain sites in the range smaller than or equal to $\bar{r}\left(\mathrm{SQ}\;\text{-}7\right)=\sqrt{10}$. In this plot, only three neighborhoods have an identical mean radius $\bar{r}\left(\mathrm{SQ}\;\text{-}3\right)=\bar{r}\left(\mathrm{SQ}\;\text{-}1,6\right)=\bar{r}\left(\mathrm{SQ}\;\text{-}1,3,6\right)=2$ (these three neighborhoods are marked by ×). Furthermore, we can empirically determine the lower limit of the percolation threshold for complex neighborhoods as

(9) $$p_{c}\left(\mathrm{SQ}\;\right)\geq \frac{p_{c}\left(\mathrm{SQ}\;\text{-}1\right)}{{\bar{r}}^{2}}.$$

This limit also holds for extended neighborhoods with sites beyond the seventh coordination zone, for example, sq-1,2,3,4,5,6,7,8, sq-1,2,3,4,5,6,7,8,9 and sq-1,2,3,4,5,6,7,8,9,10 ($p_{c}$ values for these neighborhoods are taken from Reference [73]). The results for extended neighborhoods (which are both complex and compact, marked by + in Figure 7) touch the boundary line of inequality (9).

**Figure 8:**
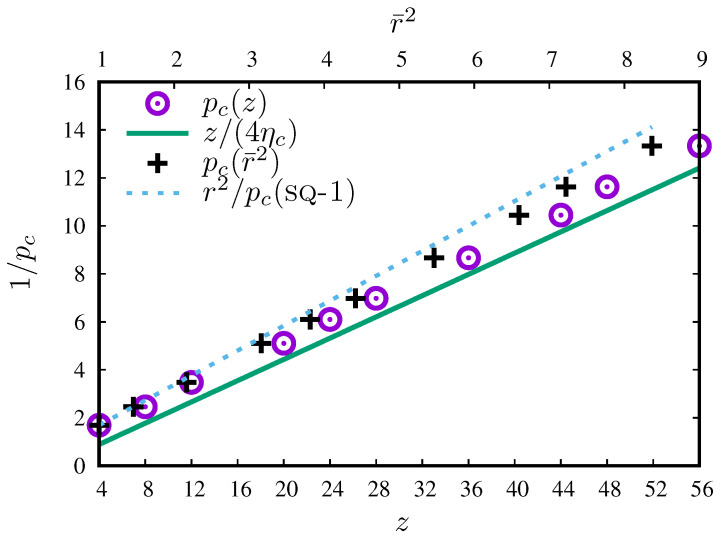
Dependence of reciprocal percolation threshold $1/p_{c}$ on ${\bar{r}}^{2}$ and *z* of the neighborhoods. Data for sq-1,2,3,4,5,6,7,8, sq-1,2,3,4,5,6,7,8,9, sq-1,2,3,4,5,6,7,8,9,10 neighborhoods are taken from Reference [73] together with the continuous percolation limit of the discs ($4\eta_{c}$).

Also in Reference [73]—from which we took values of $p_{c}$ for the sq-1,2,3,4,5,6,7,8, sq-1,2,3,4,5,6,7,8,9, sq-1,2,3,4,5,6,7,8,9,10 neighborhoods—Xun et al. studied, among other things, the percolation thresholds for regular lattices with compact extended-range neighborhoods in two dimensions. For all Archimedean lattices and up to the 10th nearest neighbors, they show the dependence *z* versus $1/p_{c}$ (Figure 7 in Reference [73]) and $-1/\ln(1-p_{c})$ (Figure 8 in Reference [73]). For new variables y=z and $x=1/p_{c}$ or $x=-1/\ln(1-p_{c})$, in both cases, the slope of the straight line close to the experimental points is y=4.521x and the value of $4.521=4\eta_{c}$ comes from the critical filling factor of circular neighborhoods in two dimensions (i.e., for the continuous percolation of discs, where $\eta_{c}=1.12808737\left(6\right)$ [88]). The experimental data for *z* vs. $1/p_{c}$ lie below this straight line as compact neighborhoods become solid discs in the limit of z→∞. In Figure 8, the reciprocals of $p_{c}$ both from our work (against $r^{2}$) and the continuous percolation limit (from Reference [73], against *z*) are presented. As we can see, for compact neighborhoods (at least for the square lattice and site percolation problem), we can confine percolation thresholds $p_{c}$ between two curves.

To conclude, we calculated 64 percolation thresholds for neighborhoods containing sites from the seventh coordination zone, of which 63 are evaluated for the first time. The obtained values of $p_{c}$ follow the early prediction of $p_{c}\left(\zeta \right)$, which is given by the power law $p_{c}\propto \zeta^{-w}$ with the exponent *w* close to 1/2. Investigating the degeneration of $p_{c}$ versus ζ allowed us to determine the lower limit $p_{c}$ as dependent on the inverse square of the mean distance $\bar{r}$ of sites in the neighborhoods. The latter touches the boundary line for the extended (compact) neighborhoods. These results enrich earlier studies of site percolation for compact neighborhoods [73] where $p_{c}$ values were restricted by the limitation predicted by $1/p_{c}>z/\left(4\eta_{c}\right)$, where $\eta_{c}$ is the critical filling factor for the continuous percolation of discs. Finally, we also recalculated $p_{c}\left(\mathrm{SQ}\;\text{-}2,4\right)=0.23288$, which means that its value provided in Reference [77], $p_{c}\left(\mathrm{SQ}\;\text{-}2,4\right)=0.225$, was clearly underestimated.

Further studies may concentrate on the estimation of the percolation thresholds $p_{c}$ for triangular or honeycomb lattices with complex neighborhoods containing sites from the seventh coordination zone or the validation of Equation (9) for other lattices.

## Figures and Tables

**Table 1:** Percolation thresholds $p_{c}$ for the square lattice with complex neighborhoods (and their characteristics *z*, ζ) containing sites from the 7th coordination zone presented in Figure 1.

Lattice	*z*	ζ	$p_{c}$
sq-1,2,3,4,5,6,7	36	84.157	0.11535
sq-2,3,4,5,6,7	32	80.157	0.11636
sq-1,3,4,5,6,7	32	78.500	0.11719
sq-1,2,4,5,6,7	32	76.157	0.11949
sq-1,2,3,5,6,7	28	66.269	0.12725
sq-1,2,3,4,6,7	32	72.844	0.12358
sq-1,2,3,4,5,7	32	72.157	0.12562
sq-3,4,5,6,7	28	74.500	0.11859
sq-2,4,5,6,7	28	72.157	0.12132
sq-2,3,5,6,7	24	62.269	0.13241
sq-2,3,4,6,7	28	68.844	0.12488
sq-2,3,4,5,7	28	68.157	0.12770
sq-1,4,5,6,7	28	70.500	0.12236
sq-1,3,5,6,7	24	60.612	0.13112
sq-1,3,4,6,7	28	67.187	0.12644
sq-1,3,4,5,7	28	66.500	0.12830
sq-1,2,5,6,7	24	58.269	0.13339
sq-1,2,4,6,7	28	64.844	0.12864
sq-1,2,4,5,7	28	64.157	0.13089
sq-1,2,3,6,7	24	54.955	0.13973
sq-1,2,3,5,7	24	54.269	0.14470
sq-1,2,3,4,7	28	60.844	0.13718
sq-4,5,6,7	24	66.500	0.12513
sq-3,5,6,7	20	56.612	0.13689
sq-3,4,6,7	24	63.187	0.12848
sq-3,4,5,7	24	62.500	0.13121
sq-2,5,6,7	20	54.269	0.13959
sq-2,4,6,7	24	60.844	0.13104
sq-2,4,5,7	24	60.157	0.13386
sq-2,3,6,7	20	50.955	0.14523
sq-2,3,5,7	20	50.269	0.19672
sq-2,3,4,7	24	56.844	0.14015
sq-1,5,6,7	20	52.612	0.13998
sq-1,4,6,7	24	59.187	0.13298
sq-1,4,5,7	24	58.500	0.13515
sq-1,3,6,7	20	49.298	0.14760
sq-1,3,5,7	20	48.612	0.14876
sq-1,3,4,7	24	55.187	0.14187
sq-1,2,6,7	20	46.955	0.14814
sq-1,2,5,7	20	46.269	0.15298
sq-1,2,4,7	24	52.844	0.14423
sq-1,2,3,7	20	42.955	0.16125
sq-5,6,7	16	48.612	0.14848
sq-4,6,7	20	55.187	0.13708
sq-4,5,7	20	54.500	0.14008
sq-3,6,7	16	45.298	0.15503
sq-3,5,7	16	44.612	0.20250
sq-3,4,7	20	51.187	0.14709
sq-2,6,7	16	42.955	0.15461
sq-2,5,7	16	42.269	0.20831
sq-2,4,7	20	48.844	0.14868
sq-2,3,7	16	38.955	0.21963
sq-1,6,7	16	41.298	0.16193
sq-1,5,7	16	40.612	0.16095
sq-1,4,7	20	47.187	0.15157
sq-1,3,7	16	37.298	0.16973
sq-1,2,7	16	34.955	0.17278
sq-6,7	12	37.298	0.17497
sq-5,7	12	36.612	0.22190
sq-4,7	16	43.187	0.16171
sq-3,7	12	33.298	0.23288
sq-2,7	12	30.955	0.23619
sq-1,7	12	29.298	0.18976
sq-7	8	25.298	0.27013

## Data Availability

Data are contained within the article or Reference [86].

## Appendix A

In Listing A1, the boundaries() procedure to be replaced in the original program published in Reference [85] is presented. The example corresponds to the sq-7 neighborhood.

**Listing A1.** boundaries() procedure for sq-7 neighborhood to be inserted in the Newman–Ziff algorithm code published in Reference [85].

## References

[1] Stauffer D., Aharony A. (1994). Introduction to Percolation Theory.

[2] Bollobás B., Riordan O. (2006). Percolation.

[3] Sahimi M. (1994). Applications of Percolation Theory.

[4] Kesten H. (1982). Percolation Theory for Mathematicians.

[5] Saberi A.A. (2015). Recent advances in percolation theory and its applications. Phys. Rep..

[6] Li M., Liu R.R., Lü L., Hu M.B., Xu S., Zhang Y.C. (2021). Percolation on complex networks: Theory and application. Phys. Rep..

[7] Sykes M.F., Essam J.W. (1964). Exact Critical Percolation Probabilities for Site and Bond Problems in Two Dimensions. J. Math. Phys..

[8] Ziff R.M., Scullard C.R. (2006). Exact bond percolation thresholds in two dimensions. J. Phys. A Math. Gen..

[9] Scullard C.R. (2006). Exact site percolation thresholds using a site-to-bond transformation and the star-triangle transformation. Phys. Rev. E.

[10] Jacobsen J.L. (2014). High-precision percolation thresholds and Potts-model critical manifolds from graph polynomials. J. Phys. A Math. Theor..

[11] Coupette F., Schilling T. (2022). Exactly solvable percolation problems. Phys. Rev. E.

[12] Akhunzhanov R.K., Eserkepov A.V., Tarasevich Y.Y. (2022). Exact percolation probabilities for a square lattice: Site percolation on a plane, cylinder, and torus. J. Phys. A Math. Theor..

[13] Broadbent S.R., Hammersley J.M. (1957). Percolation processes: I. Crystals and mazes. Math. Proc. Camb. Philos. Soc..

[14] Hammersley J.M. (1957). Percolation processes: II. The connective constant. Math. Proc. Camb. Philos. Soc..

[15] Soto-Gomez D., Vazquez Juiz L., Perez-Rodriguez P., Eugenio Lopez-Periago J., Paradelo M., Koestel J. (2020). Percolation theory applied to soil tomography. Geoderma.

[16] Bolandtaba S.F., Skauge A. (2011). Network Modeling of EOR Processes: A Combined Invasion Percolation and Dynamic Model for Mobilization of Trapped Oil. Transp. Porous Media.

[17] Mun S.C., Kim M., Prakashan K., Jung H.J., Son Y., Park O.O. (2014). A new approach to determine rheological percolation of carbon nanotubes in microstructured polymer matrices. Carbon.

[18] Ghanbarian B., Liang F., Liu H.H. (2020). Modeling gas relative permeability in shales and tight porous rocks. Fuel.

[19] Ueland B.G., Jo N.H., Sapkota A., Tian W., Masters M., Hodovanets H., Downing S.S., Schmidt C., McQueeney R.J., Bud’ko S.L. (2018). Reduction of the ordered magnetic moment and its relationship to Kondo coherence in Ce_1−x_La_x_Cu_2_Ge_2_. Phys. Rev. B.

[20] Keeney L., Downing C., Schmidt M., Pemble M.E., Nicolosi V., Whatmore R.W. (2017). Direct atomic scale determination of magnetic ion partition in a room temperature multiferroic material. Sci. Rep..

[21] Buczek P., Sandratskii L.M., Buczek N., Thomas S., Vignale G., Ernst A. (2016). Magnons in disordered nonstoichiometric low-dimensional magnets. Phys. Rev. B.

[22] Yiu Y., Bonfá P., Sanna S., De Renzi R., Carretta P., McGuire M.A., Huq A., Nagler S.E. (2014). Tuning the magnetic and structural phase transitions of PrFeAsO via Fe/Ru spin dilution. Phys. Rev. B.

[23] Grady M. (2023). Possible new phase transition in the 3D Ising model associated with boundary percolation. J. Phys. Condens. Matter.

[24] Jeong J., Park K.J., Cho E.J., Noh H.J., Kim S.B., Kim H.D. (2018). Electronic structure change of NiS_2−x_Se_x_ in the metal-insulator transition probed by X-ray absorption spectroscopy. J. Korean Phys. Soc..

[25] Avella A., Oles A.M., Horsch P. (2019). Defect-Induced Orbital Polarization and Collapse of Orbital Order in Doped Vanadium Perovskites. Phys. Rev. Lett..

[26] Cheng L., Yan P., Yang X., Zou H., Yang H., Liang H. (2020). High conductivity, percolation behavior and dielectric relaxation of hybrid ZIF-8/CNT composites. J. Alloys Compd..

[27] Xu F., Xu Z., Yakobson B.I. (2014). Site-percolation threshold of carbon nanotube fibers—Fast inspection of percolation with Markov stochastic theory. Phys. A.

[28] Sykes M., Glen M. (1976). Percolation processes in 2 dimensions. 1. Low-density series expansions. J. Phys. A Math. Gen..

[29] Sykes M., Gaunt D., Glen M. (1976). Percolation processes in 2 dimensions. 2. Critical concentrations and mean size index. J. Phys. A Math. Gen..

[30] Sykes M., Gaunt D., Glen M. (1976). Percolation processes in 2 dimensions. 3. High-density series expansions. J. Phys. A Math. Gen..

[31] Sykes M., Gaunt D., Glen M. (1976). Percolation processes in 2 dimensions. 4. Percolation probability. J. Phys. A Math. Gen..

[32] Gaunt D., Sykes M. (1976). Percolation processes in 2 dimensions. 5. Exponent *δ*_p_ and scaling theory. J. Phys. A Math. Gen..

[33] Meyers L.A. (2007). Contact network epidemiology: Bond percolation applied to infectious disease prediction and control. Bull. Am. Math. Soc..

[34] Lee D.S., Zhu M. (2021). Epidemic spreading in a social network with facial masks wearing individuals. IEEE Trans. Comput. Soc. Syst..

[35] Ziff R.M. (2021). Percolation and the pandemic. Phys. A.

[36] Malarz K., Kaczanowska S., Kułakowski K. (2002). Are forest fires predictable?. Int. J. Mod. Phys. C.

[37] Guisoni N., Loscar E.S., Albano E.V. (2011). Phase diagram and critical behavior of a forest-fire model in a gradient of immunity. Phys. Rev. E.

[38] Simeoni A., Salinesi P., Morandini F. (2011). Physical modelling of forest fire spreading through heterogeneous fuel beds. Int. J. Wildland Fire.

[39] Camelo-Neto G., Coutinho S. (2011). Forest-fire model with resistant trees. J. Stat. Mech. Theory Exp..

[40] Abades S.R., Gaxiola A., Marquet P.A. (2014). Fire, percolation thresholds and the savanna forest transition: A neutral model approach. J. Ecol..

[41] Ramírez J.E., Pajares C., Martínez M.I., Rodríguez Fernández R., Molina-Gayosso E., Lozada-Lechuga J., Fernández Téllez A. (2020). Site-bond percolation solution to preventing the propagation of *Phytophthora zoospores* on plantations. Phys. Rev. E.

[42] Rosales Herrera D., Ramírez J.E., Martínez M.I., Cruz-Suárez H., Fernández Téllez A., López-Olguín J.F., Aragón García A. (2021). Percolation-intercropping strategies to prevent dissemination of *phytopathogens* on plantations. Chaos.

[43] Herrera D.R., Velázquez-Castro J., Téllez A.F., López-Olguín J.F., Ramírez J.E. (2024). Site percolation threshold of composite square lattices and its agroecology applications. Phys. Rev. E.

[44] Cao W., Dong L., Wu L., Liu Y. (2020). Quantifying urban areas with multi-source data based on percolation theory. Remote Sens. Environ..

[45] Ng M.K.M., Shabrina Z., Sarkar S., Han H., Pettit C. (2024). From urban clusters to megaregions: Mapping Australia’s evolving urban regions. Comput. Urban Sci..

[46] Alguero M., Perez-Cerdan M., del Real R.P., Ricote J., Castro A. (2020). Novel Aurivillius Bi_4_Ti_3−2x_Nb_x_Fe_x_O_12_ phases with increasing magnetic-cation fraction until percolation: A novel approach for room temperature multiferroism. J. Mater. Chem. C.

[47] Moreira A., Andrade J., Stauffer D. (2001). Sznajd social model on square lattice with correlated percolation. Int. J. Mod. Phys. C.

[48] Malarz K., Wołoszyn M. (2023). Thermal properties of structurally balanced systems on classical random graphs. Chaos.

[49] Cirigliano L., Castellano C., Timár G. (2023). Extended-range percolation in complex networks. Phys. Rev. E.

[50] Bartolucci S., Caccioli F., Vivo P. (2020). A percolation model for the emergence of the Bitcoin Lightning Network. Sci. Rep..

[51] Beddoe M., Gölz T., Barkey M., Bau E., Godejohann M., Maier S.A., Keilmann F., Moldovan M., Prodan D., Ilie N. (2023). Probing the micro- and nanoscopic properties of dental materials using infrared spectroscopy: A proof-of-principle study. Acta Biomater..

[52] Grassberger P. (2003). Critical percolation in high dimensions. Phys. Rev. E.

[53] Sykes M.F., Essam J.W. (1964). Critical percolation probabilities by series methods. Phys. Rev..

[54] Sur A., Lebowitz J.L., Marro J., Kalos M.H., Kirkpatrick S. (1976). Monte Carlo studies of percolation phenomena for a simple cubic lattice. J. Stat. Phys..

[55] Gaunt D., Sykes M. (1983). Series study of random percolation in 3 dimensions. J. Phys. A Math. Gen..

[56] Lorenz C., Ziff R. (1998). Universality of the excess number of clusters and the crossing probability function in three-dimensional percolation. J. Phys. A Math. Gen..

[57] Kurzawski Ł., Malarz K. (2012). Simple cubic random-site percolation thresholds for complex neighbourhoods. Rep. Math. Phys..

[58] Kotwica M., Gronek P., Malarz K. (2019). Efficient space virtualisation for Hoshen–Kopelman algorithm. Int. J. Mod. Phys. C.

[59] Zhao P., Yan J., Xun Z., Hao D., Ziff R.M. (2022). Site and bond percolation on four-dimensional simple hypercubic lattices with extended neighborhoods. J. Stat. Mech. Theory Exp..

[60] Paul G., Ziff R.M., Stanley H.E. (2001). Percolation threshold, Fisher exponent, and shortest path exponent for four and five dimensions. Phys. Rev. E.

[61] Xun Z., Hao D., Ziff R.M. (2023). Extended-range percolation in five dimensions. arXiv.

[62] Van der Marck S. (1998). Calculation of percolation thresholds in high dimensions for FCC, BCC and diamond lattices. Int. J. Mod. Phys. C.

[63] Koza Z., Poła J. (2016). From discrete to continuous percolation in dimensions 3 to 7. J. Stat. Mech. Theory Exp..

[64] Haji-Akbari A., Ziff R.M. (2009). Percolation in networks with voids and bottlenecks. Phys. Rev. E.

[65] Mitra S., Saha D., Sensharma A. (2019). Percolation in a distorted square lattice. Phys. Rev. E.

[66] Mitra S., Saha D., Sensharma A. (2022). Percolation in a simple cubic lattice with distortion. Phys. Rev. E.

[67] Mitra S., Sensharma A. (2023). Site percolation in distorted square and simple cubic lattices with flexible number of neighbors. Phys. Rev. E.

[68] Cruz M.A.M., Ortiz J.P., Ortiz M.P., Balankin A. (2023). Percolation on Fractal Networks: A Survey. Fractal Fract..

[69] Dalton N.W., Domb C., Sykes M.F. (1964). Dependence of critical concentration of a dilute ferromagnet on the range of interaction. Proc. Phys. Soc..

[70] Domb C., Dalton N.W. (1966). Crystal statistics with long-range forces: I. The equivalent neighbour model. Proc. Phys. Soc..

[71] Malarz K. (2024). Universality of percolation thresholds for two-dimensional complex non-compact neighborhoods. Phys. Rev. E.

[72] Xun Z., Hao D., Ziff R.M. (2021). Site percolation on square and simple cubic lattices with extended neighborhoods and their continuum limit. Phys. Rev. E.

[73] Xun Z., Hao D., Ziff R.M. (2022). Site and bond percolation thresholds on regular lattices with compact extended-range neighborhoods in two and three dimensions. Phys. Rev. E.

[74] Xun Z., Hao D. (2022). Monte Carlo simulation of bond percolation on square lattice with complex neighborhoods. Acta Phys. Sin..

[75] Malarz K., Galam S. (2005). Square-lattice site percolation at increasing ranges of neighbor bonds. Phys. Rev. E.

[76] Galam S., Malarz K. (2005). Restoring site percolation on damaged square lattices. Phys. Rev. E.

[77] Majewski M., Malarz K. (2007). Square lattice site percolation thresholds for complex neighbourhoods. Acta Phys. Pol. B.

[78] Malarz K. (2015). Simple cubic random-site percolation thresholds for neighborhoods containing fourth-nearest neighbors. Phys. Rev. E.

[79] Malarz K. (2020). Site percolation thresholds on triangular lattice with complex neighborhoods. Chaos.

[80] Malarz K. (2021). Percolation thresholds on triangular lattice for neighbourhoods containing sites up to the fifth coordination zone. Phys. Rev. E.

[81] Malarz K. (2022). Random site percolation on honeycomb lattices with complex neighborhoods. Chaos.

[82] Malarz K. (2023). Random site percolation thresholds on square lattice for complex neighborhoods containing sites up to the sixth coordination zone. Phys. A.

[83] Privman V. (1990). Finite-Size Scaling Theory. Finite Size Scaling and Numerical Simulation of Statistical Systems.

[84] Landau D.P., Binder K. (2009). A Guide to Monte Carlo Simulations in Statistical Physics.

[85] Newman M.E.J., Ziff R.M. (2001). Fast Monte Carlo algorithm for site or bond percolation. Phys. Rev. E.

[86] Malarz K., Wołoszyn M., Ciepłucha A., Utnicki M. (2005). Lower limit of percolation threshold on a square lattice with complex neighborhoods—supplemental material. RODBUK Crac. Open Res. Data Repos..

[87] Bastas N., Kosmidis K., Giazitzidis P., Maragakis M. (2014). Method for estimating critical exponents in percolation processes with low sampling. Phys. Rev. E.

[88] Mertens S., Moore C. (2012). Continuum percolation thresholds in two dimensions. Phys. Rev. E.

